# Genetic diversity of *Venturia inaequalis* isolates (Apple scab) in China and U.K. determined by SSR markers

**DOI:** 10.1371/journal.pone.0252865

**Published:** 2021-06-10

**Authors:** Xiancheng Li, Fei Tao, Sanhong Fan, Haiyuan Li, Jiarong Yang, Liqiang Gao

**Affiliations:** 1 College of Plant Protection, Northwest A&F University, Yangling, Shaanxi, China; 2 College of Plant Protection, Gansu Agricultural University, Lanzhou, Gansu, China; 3 College of Life Sciences, Northwest A&F University, Yangling, Shaanxi, China; Sant Baba Bhag Singh University, INDIA

## Abstract

Apple scab caused by *Venturia inaequalis* is a serious disease of cultivated apple worldwide. In this study, we collected 132 *V*. *inaequalis* isolates from Shaanxi, Gansu, Xinjiang, and the U.K. and analyzed their genetic diversity by using 13 microsatellite markers. Cluster analysis based on population structure and genetic distances suggested high similarity among the four regions. Population differentiation values ranged from 0.044 to 0.155, indicating there is a high level of kinship among the four regions. All isolates could be divided into 5 lineages with a 0.76 similarity coefficient. Among the four regions, Shaanxi had only one lineage, Group II; Gansu had four lineages, Group I, Group II, Group IV, and Group V; Xinjiang had all five lineages, Group I, Group II, Group III, Group IV, and Group V; and the U.K. had three lineages, Group I, Group II and Group IV. High molecular variance was detected for populations in the four regions, with 91% of the variance occurring within the populations and 9% among the populations. Structure analysis there are three common ancestors of these four regions. The results of the present study shed light on the genetic diversity of *V*. *inaequalis* in Shaanxi, Gansu and Xinjiang, which will lead to the development of more effective management strategies and new resistant apple cultivars through molecular marker-assisted selection.

## Introduction

Apple scab caused by *Venturia inaequalis* (Cooke) G. Winter is a serious disease of cultivated apple worldwide. Without appropriate control measures, crop losses in susceptible cultivars may reach up to 70% [1]. The pathogen requires low temperatures and high humidity to initiate the sexual or ascigerous stage [2]. In addition to causing disease in apple cultivars, *V*. *inaequalis* can also infect various rosaceous ornamentals, such as crabapples, loquat, and hawthorn [3]. As most apple cultivars cannot be fertilized with their pollen, crabapples can be used as pollen sources [4].

Control strategies for apple scab has been aimed at primary ascosporic inoculum. In spring, protectant fungicides should be used to attenuate ascospore discharge and infection. In autumn, eradicant fungicides should be used to prevent pseudothecia development. A recent study revealed that ascospores contributed the most and overwintering conidia contributed approximately 20–50% as primary inoculum [5]. According to these results, reducing leaf litter in orchards has been recognized as an important strategy for controlling apple scab [6–9].

Investigations of the genetic diversity of *V*. *inaequalis* have been conducted in many regions around the world. and populations of *V*. *inaequalis* in Europe were more genetically diverse than those in other regions where apples were introduced [10–15]. A more recent study of the genetic diversity of *V*. *inaequalis* populations provided evidence that South Africa most likely has *V*. *inaequalis* subpopulations linked to diverse climatic conditions in the coastal region compared with mountainous inland regions [16]. However, populations of *V*. *inaequalis* from coastal areas in Israel are genetically uniform and populations from the Golan Heights showed levels of genotypic diversity ten times as high than coastal areas. Since sexual reproduction in this pathogen has an obligate requirement for sustained low winter temperatures and in Israel only Golan Heights meets this condition. The result indicated that *V*. *inaequalis* does not reproduce sexually in regions characterized by the absence of low winter temperatures and is instead composed of clonal lineages [17]. An study on the genetic differences among *V*. *inaequalis* isolates obtained from three different sites in Shaanxi Province of China by using simple sequence repeat (SSR) markers to analyze the structure of the disease-causing population. The result indicated that geographical factors have greater influence on genetic differences among populations than host cultivars [18]. An extensive study of genetic diversity among *V*. *inaequalis* isolates causing apple scab in 28 orchards and on five continents using 12 microsatellite markers showed that 88% of the genetic variation occurred within populations, which was consistent with extensive migration of the fungus among and within regions, while differentiation among regions was low (8% of the total variation) indicating that regions contribute a less diversity to populations [10]. In an investigation of *V*. *inaequalis* isolates in Turkey employing random amplified polymorphic DNA (RAPD), no significant differences were observed between the isolates from two different geographic regions [19]. A recent study showed that Jammu and Kashmir most likely had *V*. *inaequalis* subpopulations linked to diverse climatic conditions of the Jammu region compared to the mountainous inland Kashmir region [20]. Based on this study, the diversity of the pathogen population mainly comes from individuals. The population diversity is high in areas where the climate is favorable for sexual reproduction.

In China, the Loess Plateau, including Shaanxi and Gansu provinces, is the primary region of apple production in terms of acreage and yield, and crabapples in the Xinjiang Autonomous Region is considered to be the ancestor of modern apple cultivars. Since 1997, apple scab has been very common in the apple-growing regions of Shaanxi, Gansu and Xinjiang (part of Northwest China), where it causes serious yield losses [18]. This disease has decreased considerably in the past few years, now occurring in only some restricted areas in the Shaanxi Weibei, Gansu Qinling, and Liupanshan mountainous areas, as well as the Yili River Valley in Xinjiang, due to the cool and wet climatic conditions in summer.

The only research on the genetic variability of *V*. *inaequalis* populations in China was carried out in Shaanxi approximately ten years ago, and all isolates were collected only from this region [18, 21]. However, the details of the population structure of *V*. *inaequalis* in Northwest China remains unknown. Besides, the origin of the pathogen and the role of crabapple in the development of apple scab epidemics in Xinjiang is not fully understood. Therefore, this study aims to investigate the genetic diversity of *V*. *inaequalis* isolates collected from Shaanxi, Gansu, and Xinjiang by using microsatellite markers to determine the following: (a) whether there are genetic differences among the different regions in Northwest China; (b) whether apple scab isolates in Northwest China originate from the U.K.; and (c) the role that pathogens collected from crabapple forests in Xinjiang play in disease epidemics.

## Materials and methods

### Sampling and isolation

**Fig 1:**
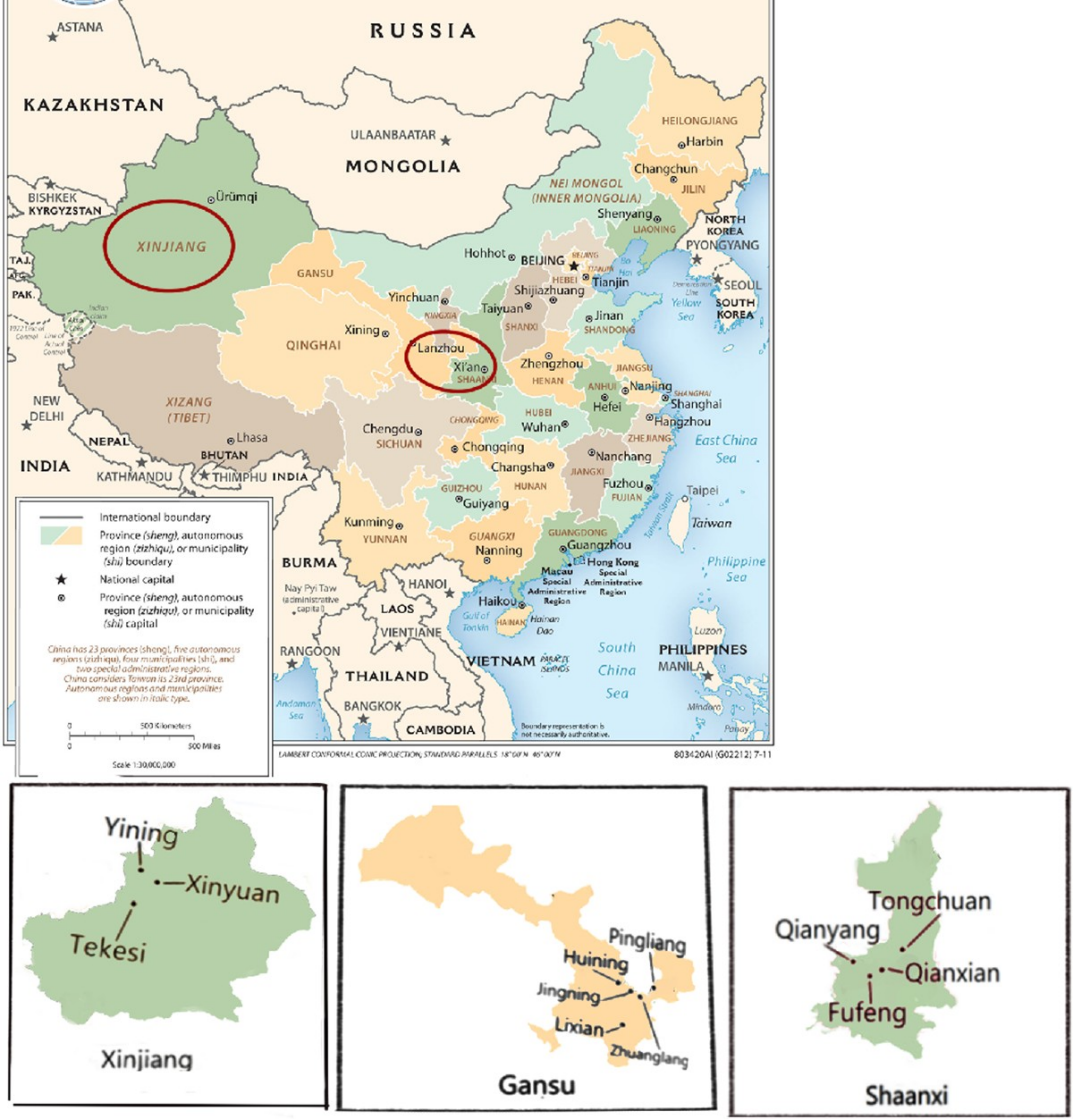
Three apple growing regions of Shaanxi, Gansu and Xinjiang in Mainland China and areas from where sample collection was carried out.

Isolates for SSR analysis were collected from four counties (Qianyang, Fufeng, Qianxian and Tongchuan) of Shaanxi Province in August 2016; five counties (Pingliang, Huining, Jingning, Lixian and Zhuanglang) of Gansu Province in September 2018; and three counties (Yining, Tekesi and Xinyuan) of the Xinjiang Autonomous Region in September 2017 (Fig 1). The counties were separated by more than 100 km, and within each county, sampled orchards were less than 1,000 m apart. Leaves displaying characteristic symptoms were collected from apple orchards and crabapple (*Malus sieversii* (Ledeb.) Roem) forests. For each infected tree, 10 leaves with obvious sporulation spots were collected for fungal isolation and considered as one sample. A cork borer with a diameter of 0.5 cm was used to cut a disc with a lesion from each leaf. Each disc was transferred to a microcentrifuge tube, air-dried at room temperature, and stored at –20°C. Water agar (15 g liter^–1^ Wokai®, China) was used to obtain single-spore isolates. An infected leaf disc was added to 1.5–2 ml of sterilized distilled water and agitated thoroughly. Approximately 200 μl of suspension was collected from water agar and spread evenly, and the moisture on the surface of the water agar was air-dried in a sterile chamber. Then, plates were incubated at 18°C for 24 h. After approximately 24 h, the individual germinated conidia in the Petri dish were marked under the microscope. Marked areas were removed with a sterile needle and placed on potato dextrose agar (PDA). The plates were storaged at 20°C, and each colony was transferred to a fresh PDA plate after 2 to 3 weeks [13]. All isolates collected from Shaanxi, Gansu and Xinjiang were designated based on the host cultivar and sampling location. Isolates collected from the U.K. were a gift from East Malling Research (September 2016) (**S1 Table**).

### DNA extraction

Mycelia of *V*. *inaequalis* isolates were inoculated on PDA medium and the medium was covered with Cellophane, stored at 20°C for 3 weeks. Approximately 30 mg of freeze-dried fungal mycelia was collected and ground in liquid nitrogen using a mortar and pestle. Then, 20 mg of the fungal powder was added to 2 mL centrifuge tubes. DNA was extracted with a Fungal DNA Extraction Kit (BioFlux®, China). The quality of DNA was checked with a NanoDrop 2000C spectrophotometer. The DNA was diluted with distilled water and stored at −20°C.

### PCR analysis

Isolates were genotyped by using 13 published SSR markers [22, 23]. The primer information is presented in Table 1.

**Table 1:** Summary of the genetic analysis results for *Venturia inaequalis* isolates according to 13 SSR loci.

Primer	Annealing Temp (°C)	Allele No.	Ne	H	I	PIC
***Vitc1/2***	58	17.00	1.56	0.36	0.54	0.85
***Vitc1/82***	58	5.00	1.30	0.23	0.39	0.29
***Vitg2/16***	58	6.00	1.85	0.46	0.65	0.54
***Viga7/116***	60	8.00	1.81	0.45	0.64	0.66
***Viga3/Z***	58	7.00	1.47	0.32	0.50	0.47
***Viaggt8/1***	58	6.00	1.40	0.28	0.46	0.30
***Vica10/154***	58	21.00	1.89	0.47	0.66	0.91
***Vitg9/99***	58	4.00	1.27	0.21	0.37	0.26
***1aac4h***	56	10.00	1.63	0.39	0.57	0.73
***1aac4b***	58	6.00	1.36	0.26	0.43	0.31
***1tc1a***	58	8.00	1.70	0.41	0.60	0.61
***EMVi001b***	55–60	14.00	1.92	0.47	0.67	0.72
***EMVi032c***	55–60	18.00	1.94	0.48	0.68	0.87

* Ne = Effective number of alleles [24]

* H = Nei’s gene diversity [25]

* I = Shannon’s information index [26]

*PIC = Polymorphism information content

For PCR amplification, a total of 25 μL of PCR mixture was prepared with 12.5 μl of 2X PCR Master Mix (reaction buffer, 4 mM MgCl_2_, 0.4 mM of each dNTPs, 0.05 U/μl Taq DNA polymerase), 1 μl of forward and reverse primers, respectively, 1 μl of DNA, adjusted to 25 μl with ddH2O. PCR reaction was performed under the following conditions: 30 cycles of 30 s at 94°C, 30 s at the specified annealing temperature (ranging from 50 to 55°C), and 20 s at 72°C. After 30 cycles, a final extension of 10 min at 72°C was performed. The PCR products were separated by Gel electrophoresis in Tris/acetic acid/EDTA (TAE) buffer solution with 3% agarose at 100 V for 1 h.

### Data analysis

The PCR-generated bands were scored as ‘1’ (presence) or ‘0’ (absence) in a binary matrix. Matrix data were imported to NTSYSpc-Version 2.1. Unweighted pair group method with arithmetic mean (UPGMA) analysis was performed with genetic distances using a simple matching coefficient. All geographic populations were analyzed to determine which populations were genetically differentiated from each other [24]. Nei’s genetic distance among the populations was estimated using POPGENE Version 1.32 and GenAlEx ver 6. 5 [25–28]. Analysis of molecular variance (AMOVA) was performed by GenAlEx 6.5 to determine the variance in allele frequencies within and among populations [29]. Individual genotypes were assigned to genetic lineages based on the Bayesian approach in STRUCTURE 2.3.4 [30]. The length of the burn-in period was set to 20000, and the number of Monte Carlo Markov chains (MCMCs) was set to 120000. The optimal K value was determined by uploading the analysis data to STRUCTURE HARVESTER (http://taylor0.biology.ucla.edu/struct_harvest/) [31].

## Results

### SSR genotypes and genetic diversity analysis

Of the 457 scab-infected sample, 124 isolates was obtained from China. In total, 132 *V*. *inaequalis* isolates were genotyped by 13 SSR markers used in the detection and all these markers were polymorphic and generated multiple alleles. The results obtained by POPGENE software for the genetic diversity parameters are presented in Table 1. A total of 130 alleles, ranging from 4 to 21 with an average of 10 per locus were detected. The locus *Vica10/154* was the most polymorphic, with 21 alleles. The effective number of alleles (Ne) ranged from 1.27 to 1.94, with an average of 1.62. Nei’s gene diversity (H) ranged from 0.21 to 0.48, with an average of 0.37. Shannon’s information index (I) ranged from 0.37 to 0.68, with an average of 0.55.

### Cluster analysis

**Fig 2:**
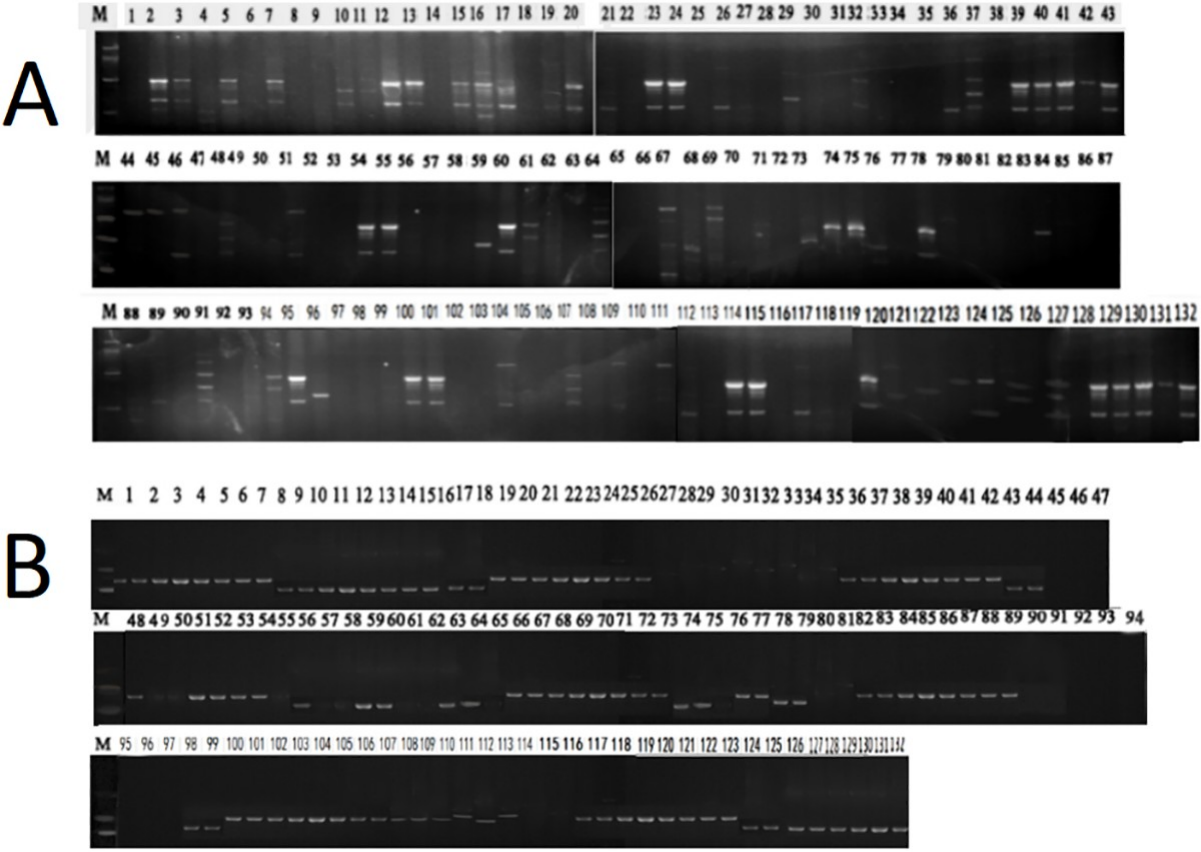
Two electrophoretograms of SSR gel. (A) Vica10/154 (B) Vitg9/99.

**Fig 3:**
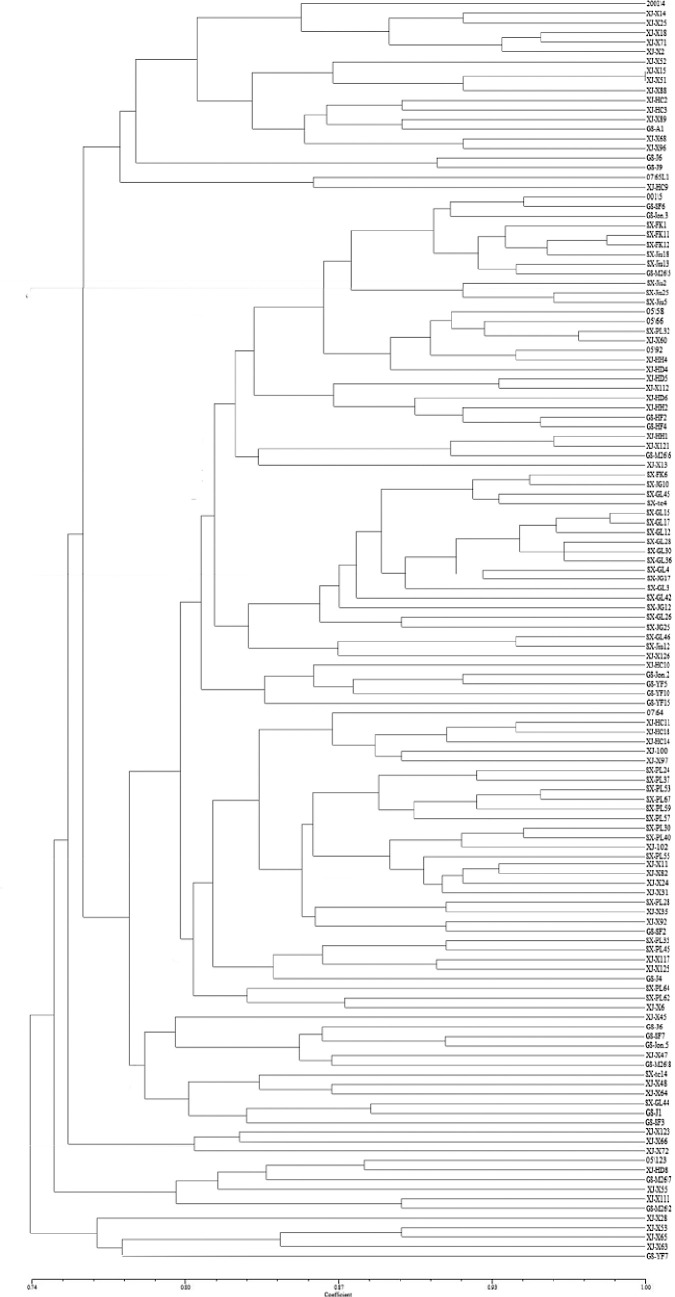
UPGMA dendrogram depiciting the genetic relationship among 132 isolates of *Venturia inaequalis* based on 13 SSR markers.

A total of 132 isolates from four regions (Shaanxi, Gansu, Xinjiang, and U.K.) were screened with 13 polymorphic SSR markers. Two electrophoretograms are shown in Fig 2. Cluster analysis of individual isolates revealed some degree of separation between the Chinese and U.K. isolates, and there was some degree of separation among the Shaanxi, Gansu, and Xinjiang isolates. The results showed no clear delineation of groups (clusters) based on location, and there were no clear indications of separation of isolates collected from different apple cultivars (Fig 3).

With a similarity coefficient of 0.76, the 132 isolates were divided into five lineages. The results are presented in Table 2. Lineages Group I, Group II, Group III, Group IV, and Group V contained 20, 98, 3, 6 and 5 isolates, accounting for 15.2%, 74.2%, 2.3%, 4.5% and 3.7% of the total number of isolates, respectively. However, populations with sample sizes less than 5 were hard to consider populations.

**Table 2:** Genetic lineages of 132 *Venturia inaequalis* isolates at a 0.76 similarity coefficient.

Region	Lineage Group/Number of Isolates				
	I	II	III	IV	V
Shaanxi	0	44	0	0	0
Gansu	3	18	0	2	1
Xinjiang	15	31	3	3	4
U.K.	2	5	0	1	0
Total	20(15.2%)	98(74.2%)	3(2.3%)	6(4.5%)	5(3.7)

The data in brackets represent the proportion of isolates in each lineage group relative to the whole population.

In terms of the number of lineages in each region (Table 3), Xinjiang had all five lineages, while Shaanxi had only one lineage (Group II), Gansu had four lineages (without Group III), and the U.K. had three lineages (without Group III and Group V). Therefore, the isolates from Shaanxi were of a single type, while those from Xinjiang were the most variable.

**Table 3:** The genetic distance and similarity of *Venturia inaequalis* populations among four different regions.

Populations	Shaanxi	Gansu	Xinjiang	UK
Shaanxi	-	0,895	0.898	0.846
Gansu	0.107	-	0.936	0.916
Xinjiang	0.104	0.066	-	0.957
U.K.	0.155	0.084	0.044	-

Notes: Genetic identity (above diagonal) and genetic distance (blow diagonal)

### Population genetic analysis

The population differentiation (Φ_PT_) values ranged from 0.044 to 0.155, indicating that between 4.4% and 15.5% of the variation occurred among the populations. Populations from the U.K. and Xinjiang were the least differentiated (Φ_PT_ = 0.044), while populations from the U.K. and Shaanxi had the highest differentiation (Φ_PT_ = 0.155) (Table 3).

### Analysis of molecular variance (AMOVA)

The lowest and highest genetic distance combination revealed population differentiation. High molecular variance was detected for populations in the four regions, where 91% of the variance occurred within populations and 9% among the populations (Table 4).

**Table 4:** Analysis of molecular variance (AMOVA) of SSR data for *Venturia inaequalis* isolates grouped by geographic origin (Shaanxi, Gansu, Xinjiang and the U.K.).

Variation source	Degrees of freedom	Sum of squares	MS	Estimated variance	Variance rate	PhiPT	P (rand > = data)
Among Pops	3	51. 438	17. 146	0. 438	9%	0.095	0.001
Within Pops	128	535. 630	4. 185	4. 185	91%		
Total	131	587. 068		4. 623	100%		

**Fig 4:**
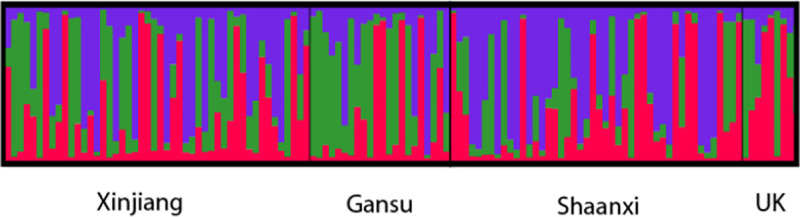
Analysis of *Venturia inaequalis* population structure shown by bar charts. Population structure showing the ancestry coefficients of *Venturia inaequalis* in Shaanxi, Gansu and Xinjiang, China, and the U.K. in the form of a bar plot (K = 3). Notes: Each vertical line represents an individual multilocus genotype. Each color represents the most likely ancestry of the cluster from which the genotype or partial genotype was derived. Individuals with multiple colors have admixed genotypes from multiple clusters.

Structure analysis divided the isolates collected from the U.K. and the 3 regions in China into 3 populations (K = 3). Some isolates from Shaanxi and Xinjiang with more long blue bars can be considered one population and to differ from others. Isolates from Gansu had the highest proportion of green bars. Isolates from the U.K. had the lowest proportion of blue bars (Fig 4).

**Fig 5:**
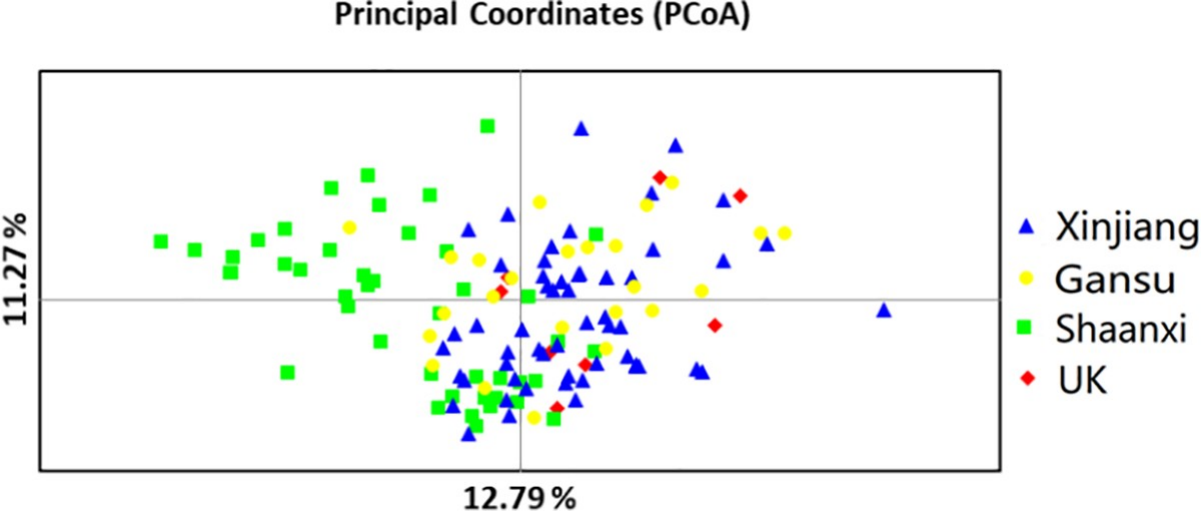
Principal coordinate analysis of *Venturia inaequalis* isolates sampled in Shaanxi, Gansu and Xinjiang, China, and in the U.K. Notes: First axis = 12.79% of total information and second axis = 11.27%.

The results of principal coordinate analysis (PCoA) showed the division of lineages intuitively (Fig 5). The symbols representing various regions exhibited a large degree of overlap, which indicated frequent gene exchange among the populations. The axes (principal axis 1 and principal axis 2) explained 12.79% and 11.27% of the variance, respectively.

## Discussion

Apple scab causes serious losses in the yield and quality of apple. The study of *V*. *inaequalis* population structure is a prerequisite for developing management strategies. The population structure of *V*. *inaequalis* in Shaanxi, Gansu, and Xinjiang, especially the origin of the pathogen and the role of crabapple in Xinjiang in the development of apple scab epidemics, is virtually unknown. We first investigated the genetic variability of *V*. *inaequalis* isolates sampled from Shaanxi, Gansu and Xinjiang in Northwest China. These results are following those of the populations from Shaanxi, Gansu and Xinjiang were highly similar. These results are in accordance with former studies in China [18, 21]. The pairwise ΦPT values of gene distance indicated that the population from Xinjiang was more similar to U.K. than other provinces. This may be related to the history of introducing apple seedlings from Europe in our sampling areas. Particularly, many EastMalling (EM) system root stocks were introduced from the U.K. to these apple-growing regions in the past [32]. As a result, the pathogen of apple scab in Shaanxi, Gansu and Xinjiang may have been introduced from the U.K. a long time ago. Besides, the wet and cool climatic conditions in the sampling areas of Gansu and Xinjiang are very favorable for disease development and may result in pathogens of *V*. *inaequalis* having a similar population structures. Our research results on genetic diversity were similar to those obtained in South Africa, indicating similar population structures of *V*. *inaequalis* under wet and cool climatic conditions [16].

The result of AMOVA showed that 91% of the variance occurred within populations and 9% among the populations, indicating geographical location had a low contribution to population differentiation. and gene flow is more frequent in these regions. The result was corresponding to many previous works [14–16]. This was credited to climate of four regions have a sustained low winter temperatures for this pathogen to product sexual reproduction makes a more genetic diversity populations. Another possible explanation of variation in assortment pressure applied by diverse cultivars.

Population stratification inferred by using cluster analyses revealed that the 132 isolates clustered into five main lineages independent of isolate sampling location and host cultivar, and in STRUCTURE 2.3.4 analysis, four region share a common three ancestry, indicating a common genetic base among the isolates. These results are in accordance with previous reports of gene flow among apple-infecting scab isolates in Switzerland [11], Pennsylvania [15] and Europe [12].

In addition, we found that isolates from crabapple in Xinjiang included all the lineages determined by the UPGMA dendrogram at a similarity level of 0.76 (Table 3). This result indicated that the migration of pathogen ascospores by wind or human-mediated transport of spores or infected plant material resulted in a mixture of genotypes. This was confirmed by more overlap among isolates from the 3 regions in China in the PCoA diagram (Fig 5). Although apple scab may be effectively controlled by fungicide applications [1], crabapple forests could serve as reservoirs for the inoculum of *V*. *inaequalis* and thus contribute to its spread, given the occurrence of the disease in crabapple and commercial apple orchards. Under climatic conditions favorable for disease development, apple scab may sweep through apple-growing areas.

Recently, new isolates that can overcome the apple scab resistance of *Malus floribunda* 821 (a resistant breeding resource) without pathogen transport from Europe to North America was found [33]. This is consistent with the finding of a previous study [34] that pathogens can overcome host resistance by evolution. Over 16 apple scab resistance genes have been found. However, most of these genes become ineffective before they are incorporated into resistance breeding [35], causing chemical agents to become the main control method. Besides, several *V*. *inaequalis* isolates with multiple fungicide resistance were also found in America [36]. All of these factors pose a serious challenge to disease control. For areas where such isolates have not been found, strict control and quarantine should be carried out. Close attention should also be paid to the population structure of the pathogen.

## Conclusion

This is the first report to use 13 microsatellite markers to measure genetic diversity in *V*. *inaequalis* populations causing apple and crabapple scab in Shaanxi, Gansu, and Xinjiang of Northwest China and the U.K. Our results showed that populations were highly similar among the four regions and indicated that 132 *V*. *inaequalis* isolates may share a common three ancestry. Our results revealed for the first time that crabapple forests could serve as inoculum reservoirs of the pathogen *V*. *inaequalis*, contributing to its spread to commercial apple orchards. Our study is a basic assay to improve the disease management strategies for the management of apple scab.

## Supporting information

S1 Table Detail information of the geographic location, apple cultivars were collected and used for genotyping. (DOCX)

S1 Dataset (RAR)
