# Supplementary material for: Genetic diversity of Venturia inaequalis isolates (Apple scab) in China and U.K. determined by SSR markers
Source: PLoS One. 2021 Jun 10;16(6):e0252865. doi: 10.1371/journal.pone.0252865 (PMC8191898; doi:10.1371/journal.pone.0252865)
Supplement: S1 Table — (DOCX) [file pone.0252865.s001.docx]

**S1 Table. Detail information of the geographic location, apple cultivars were collected and used for genotyping.**

| **Sampling region** | **Sampling country** | **Isolate code** | **No. isolates collected** | **Cultivar** |
| --- | --- | --- | --- | --- |
| Xinjiang | Xinyuan | XJ-X100, XJ-X102, XJ-X11, XJ-X111, XJ-X112, XJ-X117, XJ-X121, XJ-X123, XJ-X125, XJ-X126, XJ-X13, XJ-X14, XJ-X15, XJ-X18, XJ-X2, XJ-X24, XJ-X25, XJ-X28, XJ-X31, XJ-X35, XJ-X45, XJ-X47, XJ-X48, XJ-X51, XJ-X52, XJ-X53, XJ-X55, XJ-X6, XJ-X60, XJ-X63, XJ-X64, XJ-X65, XJ-X66, XJ-X68, XJ-X71, XJ-X72, XJ-X82, XJ-X88, XJ-X89, XJ-X92, XJ-X96, XJ-X97 | 42 | Crabapple |
|  | Yining | XJ-HH1, XJ-HH2, XJ-HH4 | 3 | Huahong |
|  |  | XJ-HD4, XJ-HD5, XJ-HD6, XJ-HD8 | 4 | Huadan |
|  | Tekesi | XJ-HC10, XJ-HC11, XJ-HC14, XJ-HC18, XJ-HC2, XJ-HC3, XJ-HC9 | 7 | Honeycrisp |
| Gansu | Jingning | GS-SF2, GS-SF3, GS-SF6, GS-SF7 | 4 | Fuji |
|  |  | GS-Jona2, GS-Jona3, GS-Jona5 | 3 | Jonagold |
|  | Huining | GS-M26\2, GS-M26\5, GS-M26\6, GS-M26\7, GS-M26\8 | 5 | Fuji |
|  | Pingliang | GS-YF10, GS-YF15, GS-YF5, GS-YF7 | 4 | Yanfu |
|  | Zhuanglang | GS-HF2, GS-HF4 | 2 | Hanfu |
|  | Lixian | GS-J1, GS-J16, GS-J4, GS-J6, GS-J9 | 5 | Fuji |
|  |  | GS-A1 | 1 | Ariane |
| Shaanxi | Qianyang | SX-GL15, SX-GL17, SX-GL26, SX-GL3, SX-GL28, SX-GL12, SX-GL30, SX-GL36, SX-GL4, SX-GL42, SX-GL44, SX-GL45, SX-GL46 | 13 | Gala |
|  |  | SX-FK1, SX-FK11, SX-FK12, SX-FK6 | 4 | Fuji |
|  |  | SX-JG10, SX-JG12, SX-JG17, SX-JG25 | 4 | Gold delicious |
|  | Fufeng | SX-Jiu12, SX-Jiu13, SX-Jiu18, SX-Jiu2, SX-Jiu25, SX-Jiu5 | 6 | Jiuyueqiji |
|  | Qianxian | SX-PL24, SX-PL28, SX-PL30, SX-PL32, SX-PL35, SX-PL37, SX-PL40, SX-PL45, SX-PL53, SX-PL55, SX-PL57, SX-PL59, SX-PL62, SX-PL64, SX-PL67 | 15 | Pink lady |
|  | Tongchuang | SX-TC4, SX-TC14 | 2 | Fuji |
| U.K. | Kent | 2001\4, 001\5, 05\123, 05\58, 05\66, 05\92, 07\64, 07\65L1 | 8 | Unknown |
